# A novel angiotensin-I-converting enzyme inhibitory peptide from oyster: Simulated gastro-intestinal digestion, molecular docking, inhibition kinetics and antihypertensive effects in rats

**DOI:** 10.3389/fnut.2022.981163

**Published:** 2022-08-23

**Authors:** Hui Chen, Yu Chen, Huizhen Zheng, Xingwei Xiang, Lu Xu

**Affiliations:** ^1^College of Food Science and Technology, Zhejiang University of Technology, Hangzhou, China; ^2^Key Laboratory of Marine Fishery Resources Exploitment and Utilization of Zhejiang Province, Hangzhou, China; ^3^Collaborative Innovation Center of Seafood Deep Processing, Dalian Polytechnic University, Dalian, China; ^4^Zhejiang Marine Fisheries Research Institute, Zhoushan, China; ^5^Key Laboratory of Sustainable Utilization of Technology Research for Fishery Resource of Zhejiang Province, Zhoushan, China; ^6^National R&D Branch Center for Pelagic Aquatic Products Processing (Hangzhou), Hangzhou, China; ^7^Department of Animal Medicine, College of Agriculture and Forestry Science, Linyi University, Linyi, China

**Keywords:** angiotensin-I-converting enzyme (ACE), oyster peptides, hypotensive, molecular docking, simulated gastro-intestinal digestion

## Abstract

In this study, a novel peptide, AEYLCEAC with high angiotensin-I-converting enzyme inhibitory (ACEI) activity was screened from oyster (Crassostrea gigas) hydrolysates, which was obtained from simulated gastro-intestinal digestion. Candidate peptides were confirmed to have a higher binding to angiotensin-I-converting enzyme (ACE) than the positive drug phosphoinic tripeptide calculated by Discovery Studio, and AEYLCEAC showed the highest ACE inhibition rate *in vitro* with a *IC*_50_ of 4.287 mM. Lineweaver-Burk plots confirmed that the peptidic inhibitory type of ACE is competitive. The molecular docking showed that ACEI activity of the AEYLCEAC was mainly due to the hydrogen bonding interactions with the active pockets (S1 and S2) of ACE. *In vivo*, AEYLCEAC effectively reduced diastolic blood pressure (DBP) and Systolic blood pressure (SBP) in hypertensive rats. These results indicate that AEYLCEAC might act as a helpful ingredient in functional foods or pharmaceuticals for the prevention and treatment of hypertension.

## Introduction

Cardiovascular disease (CVD) has been recognized as the leading cause of mortality globally (1). Hypertension has typical characteristics of age-related diseases which are considered to be the main risk factor associated with cardiovascular disease CVDs (2). The Angiotensin I-converting enzyme (ACE) plays a significant role in blood pressure regulation *in vivo* through the renin-angiotensin system (RAS) along with the kallikrein-kinin system (KKS). In the RAS, the active vasopressor angiotensin II (Ang II) could be released by cleavage of C-terminal dipeptides from the inactive angiotensin I (Ang I) catalysis by ACE. Meanwhile, within the KKS, ACE could also degrade the bradykinin (BK) after the removing two C-terminal dipeptides (1). Ang I is a vasopressor substance and BK acts as a hypotensive oligopeptide, ACE inhibition is deemed to be an effective way for hypertension prevention and treatment.

Many synthetic ACE inhibitors like captopril, enalapril, benazepril and lisinopril have been widely used in the clinical prevention of hypertension (3). However, frequent intake of these drugs will cause serious adverse effects such as headache, dry cough, hypotension, taste disturbances, allergic reactions, hyperkalemia, renal failure, angioedema and fetal abnormalities (4). Hence, the search for safer ACE inhibitors with minimal side effects always in the spotlight. It is generally known that ACE inhibitory peptides obtained from natural food rarely cause adverse side effects and therefore have the potential for further utilization. Previous research have proved that these peptides exhibit a variety of physiological functions including antimicrobial, antihypertensive, immunomodulatory, antithrombotic, antioxidant, hypolipidemic, and anticancer effects (5). Up to now, a large of ACEI peptides have been found derived from milk, eggs, rice, whey protein, cod skin and marine shellfish (6, 7). Oyster is an abundant marine resource with high protein content, and oyster peptides have also been reported to have an antihypertensive activity. Shiozaki et al. isolated an effective ACEI peptide from oyster trypsin hydrolysate (8). Wang et al. also identified peptides from oyster protein hydrolysate which exhibit antihypertensive effect (9). The functional activity of these peptides depends mainly on their sequence and structure, and the biological efficacy of peptides is determined by their bioavailability to a large extent. The main reason for the low bioavailability of the peptides is the instability during gastrointestinal (GI) digestion, selective transport, and plasma peptidase degradation, which are important factors that severely limit their antihypertensive effects (10, 11). Biopeptides need to remain active after digestion and be transported through the intestinal wall into the bloodstream before they can play an antihypertensive role *in vivo* (12). The peptides digested by pepsin and trypsin are inclined to have stronger enzyme tolerance in the gastrointestinal tract and are more capable of performing their biological functions. In our previous study, antithrombic peptides of oyster (Crassostrea gigas) released from the simulated gastroenteric digestion were screened using UPLC-QTOF and molecular docking, which also are tolerance to digestive enzyme (13). However, few articles were reported that the hydrolysate of simulated digestion from oysters can inhibit ACE.

Various spectroscopy methods are widely used to reveal molecular interactions, such as spectrophotometers, fluorescence spectroscopy, etc., circular dichroism (CD) (14–17). In addition, molecular docking, a simulation algorithm is also used to calculate the physical and chemical properties, spatial structure, and biological activity of protein or peptides (18–20). Based on the lock and key principle of ligand and receptor, molecular docking can calculate the detailed interaction information between receptor and ligand, to screen out potentially effective ACEI peptides (21). Due to the elucidation of the crystal structure of the angiotensin-converting enzyme and its inhibitor complex in RCSB PDB,^1^ molecular docking generally uses protein crystal structure as the enzyme-inhibitor template for the molecular discovery and mechanism analysis of ACEI peptides.

This study aims to screen a novel ACEI peptide sequence from oyster hydrolysates in simulated digestion via molecular docking simulation. And the antihypertensive effects of the chemically synthesized peptides were verified *in vitro* and *in vivo*. What’s more, the *IC*_50_ value of the target peptide was calculated, and the potential ACEI kinetics of the peptide were investigated by the Lineweaver-Burk plots. Besides, the structure-activity relationship and binding interactions of the screened peptide with the active site of ACE were elucidated through molecular docking. The interactions between the peptide and ACE were further verified by CD spectroscopy and fluorescence spectroscopy as well.

## Materials and methods

### Materials and chemicals

Briefly, the oyster peptide was obtained by simulated gastric digestion for 2 h and then simulated intestinal digestion for 3 h, which has been reported previously (13). After obtained, the samples were stored at −20°C until for use. Angiotensin-I converting enzyme (ACE) (from rabbit lungs), Hippuryl-histidyl-leucine (HHL), acetonitrile (ACN), and trifluoroacetic acid (TFA) were purchased from Sigma-Aldrich Co. (St. Louis, MO, United States). All other chemical reagents were of analytical or HPLC grade.

### Molecular docking screening of the potential angiotensin-I-converting enzyme inhibitory peptides

*In silico* methods were adopted to screen the potential ACEI peptides. The affinity between peptides and ACE was evaluated by molecular docking. All the peptidic molecules were docked with ACE using the CDOCKER program in Discovery Stidio software to screen for potential ACEI peptides. The native crystal structure of human ACE (PDB ID: 4CA5) was downloaded from the PDB protein database (see text footnote 1). Before docking, water molecules in conformation were removed, and the protein was hydrogenated to further optimize the 3D-structure. The 3D-conformation with minimum energy of the target peptides was calculated. The force types between the ligand and the docked receptor were also analyzed. The potent binding peptides to the ACE were determined based on the -CDOCKER ENERGY score (21).

### Chemical synthesis of peptides

The potential ACEI peptides predicted by molecular docking analysis were ultimately synthesized at Chinapeptides Limited Corporation (Shanghai, China) via a solid-phase synthesis method. All the synthetic peptides are greater than 95% pure according to HPLC.

### Identification of the synthesized peptides

Isolation of the peptides was performed on an RP-HPLC system equipped with a C18 column (Kromasil, 150 × 4.6 mm, 3.0 μm, Luna SU Phenomenex, Torrance, CA, United States). The mobile phase consisted of 0.1% TFA in acetonitrile (buffer A) and 0.1%TFA in water (buffer B). The gradient elution was performed at 30°C with 53–78% buffer B at a flow rate of 1.0 mL/min throughout the separation. The gradient elution operated as follows: 0–5 min: 5% buffer B; 5–20 min: 5–50% buffer B; 20–30 min: 50–85% buffer B; 30–35 min: 85–5% buffer B. The injection volume was 10 μL, and the peptide concentration was about 1.0 mg/mL. The MS spectra were obtained by positive electrostatic ionization. The ESI source parameters were a dry temperature of 200°C, a dry gas speed of 9.0 L min^–1^, a capillary voltage of + 3 kV, and a 1.8 bar nebulizer gas. The collision energy was regulated from 23 to 65 eV as a function of the m/z value. The LC system was connected to a quadrupole time-of-flight (Q-TOF) spectrometer using an electrospray ionization source (ESI; Bruker, Germany). The initial calibration target of the ESI-Q-TOF mass spectrometer is the peptide chains in the mass range of 300–2,000 m/z. The sequence of peptides were analyzed by *de novo* sequencing and searched by Mascot (Version 2.4, Matrix Science Inc., London, United Kingdom).

### Assay of the angiotensin-I-converting enzyme-inhibitory activity

The ACEI activity of the synthesized peptides was measured following the reported method with a minor modification (22). The experiment was carried out in a 1.5 mL PE tube. Briefly, for each assay, 20 μL of the inhibitor (1 mg/ml) was mixed with 120 μL of the HHL solution in the concentration of 5 mM. The mixture was first preincubated at a constant temperature of 37°C for 5 min, 10 μL ACE (0.1 U/mL) was then added to start the reaction, and to incubate at 37°C for 60 min. In the end, 150 μl HCl (1M) was used to terminate the reaction. Meanwhile, the inhibitor solution was replaced with 20 μL boric acid buffer solution (0.1 mol/L, pH 7.3, containing 0.3 mol/L NaCl) as a blank control group. The HPLC system was equipped with a ZORBAX Eclipse SB-C18 column (5 μm particle size, 4.6 × 150 mm). To analyze the releasing amount of hippuric acid under the action of ACE, the elution was conducted in 25% Mobile Phase A (ACN) and 75% Mobile Phase B (0.05% TFA in ultrapure water) for 25 min with a flow rate of 0.8 mL/min. The ACE inhibitory activity percentage (%) was calculated based on the equation below:


ACEinhibitionrate(%)=(B-S)/B×100

Where B and S are the chromatographic peak areas of the control group and the sample tested, respectively. *IC*_50_ is the inhibitor concentration capable of inhibiting 50% of ACE activity under the described experimental conditions, which was estimated by non-linear regression of ACE inhibition (%) against different inhibitor concentrations by using SPSS software.

### Kinetic parameters of angiotensin-I-converting enzyme inhibition

To explore the inhibition pattern of the peptide and clarify the kinetics of ACEI activity, the Lineweaver-Burk plot was employed in our study. The various concentrations of the HHL solution were 1, 2, 5, 8, and 10 mM, and the concentration of synthesized peptide was set as 2 mg/mL. Various concentrations of HHL and peptide were incubated in ACE solutions. The Y-intercept of the Lineweaver-Burk plot represents the reciprocal of the maximum velocity (*Vmax^–1^*), while the X-intercept is the reciprocal of the Michaelis-Menten constant (*Km^–1^*).

### Circular dichroism spectrometry

CD spectroscopy was adopted to investigate the ACE conformation before and after the addition peptide. The experiments were conducted by a Jasco815 spectropolarimeter (Jasco, Tokyo, Japan), with a wavelength range of 190–260 nm. The experiment of CD was conducted based on the method reported by Memarpoor-Yazdi et al. (23). In brief, a concentration of 0.12 mg/mL ACE was dissolved in Tris-HCl (50 mM, pH 7.5), with 0.03 mM NaCl. The concentration of the screened oyster peptide in the ACE buffer was 0.02 mg/mL. The spectra of ACE and oyster peptide were measured at concentrations of 0.06 mg/mL and 0.01 mg/mL, respectively. In the presence of the peptide, the spectra of ACE were measured at an ACE: peptide molar ratio of approximately 7:1. The solution of ACE (0.07 mg/mL) was used as the blank control. Calibrate the baseline by subtracting the buffer spectrum in all measurements. The fractions of secondary structure elements were evaluated using software such as SELCON3 (24), CDSSTR (25), and CONTIN (25).

### Fluorescence spectrometry analysis

Fluorescence spectra of ACE reacting with different concentrations of the synthetic peptides (1.1 mM, 0.58 mM, and 0.29 mM) were acquired using the SYNRGY H1 microplate reader (BioTek, United States), and black 96-well plates were used. Fluorescence intensities were corrected for inner filter and dilution effects before analysis of the quenching data. After equilibration for five min, the fluorescence spectra were determined at a wavelength range of 300–700 nm with an excitation wavelength of 270 nm, while the scanning rate was 120 nm/min. Both excitation and emission slit widths were fixed to 2 nm. Three copies were scanned for each test and the baseline correction with ACE buffer was utilized as a blank control (26).

### Animal trial

Male SHRs aged 10–12 weeks old, body weight (BW) of 220–250 g, and blood pressure over 180 mmHg were purchased from Trophic Animal Feed High-tech Co. Ltd., (Nantong, China). Six SHRs were administrated individually in each cage with the stable room temperature (25 ± 0.5°C), the humidity of 55 ± 5%, and a 12-h light to12-h dark cycle. Food and water were freely accessed. Rats were accustomed to the above environment for 2 weeks before the experiment. The experiments were conducted in accordance with the National Institutes of Health Guide for the Care and Use of Laboratory Animals (NIH Publications No. 8023, revised 1978). And all equipment and experimental procedures were approved by the Ethics Committee on Animal Experimentation of Zhejiang University of Technology (Permission Number 20210309043). The SHRs were randomly grouped in 3. Rats were gavage administered with peptide at 15 mg/kg⋅BW/day in the experiment group. Control group was given water, and the positive group was gavaged with captopril at 10 mg/kg⋅BW/day. All rats were gavaged with their daily dose at 8 am, and the blood pressure was measured every 3 h by 4 times, which was using the tail-cuff method (27). Briefly, rats were covered with a fixed device but could let their tail leak out (MK2000ST, Muromachi Kikai Co., Ltd., Japan). A cuff was curled around the tail to measure the diastolic blood pressure (DBP) and systolic blood pressure (SBP).

In a long-term experiment, SHRs were randomly grouped in 3 as above. The negative, positive control, and peptide group were carried out also the same as above, but all rats were administered at 15 mg/kg⋅BW/day for 4 weeks. Daily administration was gavaged at 8 a.m., while the SBP and DBP were measured using the tail-cuff method weekly at 2 p.m. during a 4-week feeding trial. Afterward, all rats were executed by 30 mg/kg pentobarbital anesthesia. Blood was collected from the aorta ventralis and the plasma was gathered at 3,000 g centrifuged for 30 min. Kidneys were taken at the end of the animal experiment and preserved with liquid nitrogen.

### RT-PCR

RNA of rats was extracted and the total mRNA reverse transcription to cDNA was performed by a standard process (PrimeScript^Tm^ RT reagent Kit with gDNA Eraser, Takara, Dalian, China). Six reference genes were designed based on the United States National Center for Biotechnology Information (NCBI) database or reported data. Primer sequences were cross-checked and synthesized by Sangone Biotech., China. RT-PCR Reaction system was carried out using the kit method (28) of TB Green^Tm^ Premix Ex Taq^Tm^ II (Tli RNaseH Plus, Takara). Quantitative PCR was performed on qTower 2.2 system (AXYGEN, CA, United States). Primer list of β-Actin (Actb), Renin 1 structural (Ren1), Angiotensin I converting enzyme (peptidyl dipeptidaseA) 1 (Ace), Angiotensin II receptor type 1b (Agtr1b), Adrenergic receptor β3 (Adrb3) was listed in Supplementary Table 1.

### Statistical analysis

The tests were carried out in triplicate (except for the molecular docking simulation results), the data were represented as the mean ± SD deviations from the triplicates. SPSS 11.5 statistical software (IBM Inc., Chicago, IL, United States) was used to evaluate the significant differences in this study. The values were considered significant with a confidence limit of 95% (*p < 0.05), 99% (**p < 0.01).

## Results and discussion

### Angiotensin-I-converting enzyme inhibitory peptides screening from oyster protein hydrolysates

In the previous study, we identified the peptide sequence of oyster hydrolysates released via simulated gastrointestinal digestion (13) The results are listed in Supplementary Table 2. In general, most of the ACE inhibitory peptides typically contain 2–12 amino acid residues (29). According to some research the peptides with short length display ACEI activity to some extent, which may reflect the presence of a small groove in ACE that prevents larger peptides from entering the active site (30). The active peptide must be able to resist the effects of gastrointestinal digestion so that it can remain bioactive after being absorbed through the intestinal epithelium. To screen the ACEI peptide, the molecular docking method was used in this study to evaluate their affinity with ACE. The peptides docking with the active center of targets, and the -CDOCKER ENERGY is calculated using Discovery Studio. The -CDOCKER ENERGY score is used to predict the stability of the peptides-targets connection, which indicates the affinity of the peptides and ACE. The higher the energy of -CDOCKER ENERGY, the more stable the ligand can bind with the receptor and obtain a more favorable conformation. As shown in Table 1, seven potential ACEI peptides, AEYLCEAC, RSNDGPI, VILGDADLP, TNEVEGPS, LDVSWASD, QEVVMGEC, and LSSNLHG exhibited energy scores higher than that of the phosphinic tripeptide, a native ligand (134.512 kcal mol^–1^). And peptide AEYLCEAC has the strongest affinity to ACE with a -CDOCKER ENERGY score of 170.423 kcal mol^–1^, which was therefore predicted to have the best ACEI activity among all identified sequences.

**TABLE 1:** The -CDOCKER_ENERGY scores and lengths of the potential ACEI peptides analyzed by molecular docking.

No.	Peptides	Length	-CDOCKER ENERGY (kcal mol^–1^)
1	phosphinic tripeptide	3	134.512
2	RSNDGPI	7	144.98
3	AEYLCEAC	8	170.423
4	VILGDADLP	9	171.449
5	TNEVEGPS	8	163.144
6	LDVSWASD	8	167.367
7	QEVVMGEC	8	160.843
8	LSSNLHG	7	169.644

**FIGURE 1:**
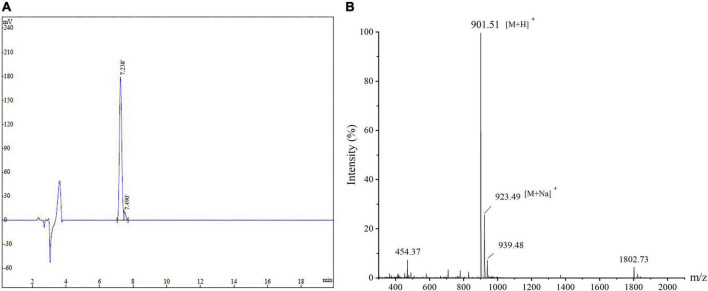
The amino acid sequence and molecular mass identification of peptide AEYLCEAC by **(A)** HPLC and **(B)** MS.

In order to verify the antihypertensive effects of these peptides, all pepides are synthesized with verificate by MS are shown in Supplementary Figure 1. In addition, their ACE inhibition rates were measured and listed in Table 2. It was also found that AEYLCEAC has the best inhibitory effect on ACE compared with several other peptides. The ACE inhibition rates of AEYLCEAC were 14.29 and 29.33% at 1 mg/mL, and 2 mg/mL AEYLCEAC, respectively. Therefore, AEYLCEAC was predicted to be an efficient ACEI peptide. The UPLC and MS results of the predicted peptide AEYLCEAC were shown in Figure 1.

**TABLE 2:** The ACE inhibition rates of seven potential ACEI peptides at different concentrations (1 and 2 mg/mL).

No.	Peptides	ACE inhibition rate (%)	
		Concentration (1 mg/mL)	Concentration (2 mg/mL)
1	RSNDGPI	–13.9647	–4.3447
2	AEYLCEAC	14.2857	29.3266
3	VILGDADLP	1.6051	4.7219
4	TNEVEGPS	–4.8796	7.9218
5	LDVSWASD	3.6918	13.9971
6	QEVVMGEC	–3.8523	–5.0688
7	LSSNLHG	–1.1236	–5.7205

### Angiotensin-I-converting enzyme-inhibitory activity determinations

**FIGURE 2:**
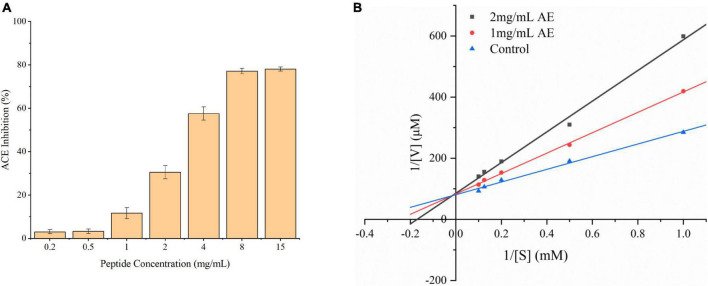
**(A)** ACE-inhibitory activity of the peptide AEYLCEAC determined by HPLC. The *IC*_50_ value was calculated by regression equation: Y = −0.0073X^2^ + 0.1616X-0.0105 (*R^2^* = 0.99). **(B)** Line weaver-Burk plot of ACE inhibition by AEYLCEAC. The ACE activity was measured with various concentrations of the peptide AEYLCEAC. (■: 2 mg/mL AEYLCEAC; •: 1 mg/mL AEYLCEAC; ▲: control).

The ACEI activity of the synthesized peptide (AEYLCEAC) with different concentrations (0.2–15 mg/mL) was determined by the HPLC method, shown in in Figure 2A. The potency of these marine-derived peptides to inhibit ACE activity has been expressed as an *IC*_50_ value, which is the ACE inhibitor concentration leading to 50% inhibition of ACE activity. When comparing with the *IC*_50_ values of previous studies. The regression equation between the ACEI activity (y) and peptide concentration (x) was calculated: y = −0.0073x^2^ + 0.1616x −0.0105 (*R^2^* = 0.99), and the *IC*_50_ value was 4.29 mM determined by logarithmic linearization (6). Almost all the results suggested that the lower The *IC*_50_ was, the more beneficial it was to reduce SBP and DBP in hypertensive rats. In recent years, *in vitro* the ACE inhibitory activity of oyster hydrolytic peptides has received increasing attention. It is known that the molecular weight, hydrophobicity as well as the composition and sequence length of the peptide are the major factors that determine its inhibitory activity (12). In this experiment, peptide AEYLCEAC obtained through GI digestion has eight amino acid residues, which is smaller than that of some high ACEI active peptides reported before (10, 31, 32). AEYLCEAC is consistent with previous findings which concluded that short peptides with low molecular mass may have higher ACEI activity (33, 34). Cheung et al. (35) suggest that peptides containing higher hydrophobic amino acids may have better ACE inhibitory activity, for the hydrophobicity of peptides can effectively enhance ACEI activity. In this study, AEYLCEAC peptide contains the hydrophobic amino acids of Ala and Leu, which may be the reason for its ACEI activity. In addition, the HAA residue Leu plays a vital role in inhibiting ACE activity, which could enhance the ACE inhibitory potential (36). Leu-containing peptides, such as VGLPNSR, QAGLSPVR, IPALLKR and AQQLAAQLPAMCR, have been reported as ACE inhibitors in many studies (37, 38). It has also been reported that ACE binding ability was largely affected by acidic amino acids including Asp and Glu. AEYLCEAC exhibited Glu at the second position of the N-terminus, which may be responsible for ACE inhibition. And it has been acknowledged that especially the C-terminal tripeptide sequence play an important role in inhibiting ACE activity. And the N-terminal rich in branched-chain aliphatic amino acids possibly led to higher ACEI activity (39, 40). Therefore, the inhibition of ACE activity may be partly due to the presence of aliphatic amino acid Ala located at the N-terminal of AEYLCEAC. In conclusion, the ACE inhibitory activity of AEYLCEAC may be partly related to the presence of C-terminal tripeptide sequence (Glu-Ala-Cys), N-terminal hydrophobic aliphatic amino acid (Ala), and the HAA residue (Leu).

### Determination of the angiotensin-I-converting enzyme-inhibitory pattern

ACEI peptides can directly inhibit ACE activity through binding to the active center in key amino acid residues, so that prevent the substrate from binding to ACE by competing with the substrate mediately. The inhibitory patterns of the ACEI peptides mainly contain competitive inhibition, non-competitive inhibition, and mixed inhibition according to their various structures. For example, competitive ACEI peptides, namely ALVY from Gac Seed Protein Hydrolysates, PRY derived from potato patatin, and SSYYPFK from naked oat globulin hydrolysates, have been published (41, 42). Non-competitive ACE inhibitors, such as VGLPNSR and QAGLSPVR, have been reported from tilapia skin gelatin, AVKVL, YLVR, and TLVGR derived from hazelnut (Corylusheterophylla Fisch), and ETSGMKPTEL and ISSMGILVCL isolated from longan seeds (32, 37). Besides, a few peptides, such as NMAINPSKENLCSTFCK isolated from casein hydrolysates, EPNGLLLPQY, QLVP from walnut protein and Mycelia of Ganoderma Lucidum (Agaricomycetes) respectively, exhibited inhibition in a mixed pattern (43, 44).

The Lineweaver-Burk plots were employed to elucidate the ACE inhibition kinetic of the synthetic peptide AEYLCEAC, shown in Figure 2B. Whereas the *V*_*max*_ was the same when the concentrations of the inhibitory peptide increased, the *Km/V_*max*_* value increased. This suggests that AEYLCEAC is a competitive inhibitor of ACE, and may affect the conformation of the ACE enzyme in the active site. Competitive inhibitors generally have a similar interactions to the enzymes and can compete with substrates for the active sites, preventing the bonding of substrate to enzyme. Among all the ACE inhibition patterns, competitive inhibition is much more common. The clinical drugs (lisinopril, captopril, and enalapril) currently used for hypertension treatment are all competitive inhibitors, which have strong ACEI activity by contending with substrates and binding to ACE active sites (30).

### Effect of AEYLCEAC on blood pressure in hypertensive rats and related genes expression

**FIGURE 3:**
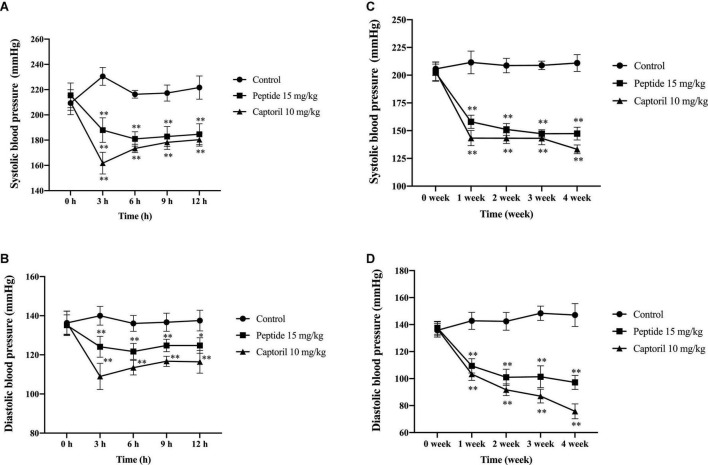
Effect of AEYLCEAC on blood pressure of SHRs’ rats after single and continuous administration. **(A)** SBP after a single intragastric administration to all groups. **(B)** DBP after a single intragastric administration to all groups. **(C)** SBP after continuous intragastric administration to all groups. **(D)** DBP after continuous intragastric administration to all groups. All data are present as the mean ± SE. *p < 0.05, ^**^p < 0.01.

For the As shown in Figure 3A, in a 12-h animal trial, SBP in control group was significantly higher than that in the peptide group and positive group after the administration from 3 to 12 h (**p < 0.01). And both SBP and DBP is lowest in at 3 h, then increased slightly. Compared with the peptide group, DBP in the peptide group was significantly decreased (^**^p < 0.01). In Figure 3B, in a 4-week of animal trial, both SBP and DBP in the peptide group were significantly decreased from 1 to 4 weeks (**p < 0.01) while the DBP of control group increased at 4 week significantly shown in Figures 3C,D. All the data of rat blood pressure change indicated that peptide group can reduce blood pressure of rats with hypertension, though the effect of Captoril was better than AEYLCEAC.

**FIGURE 4:**
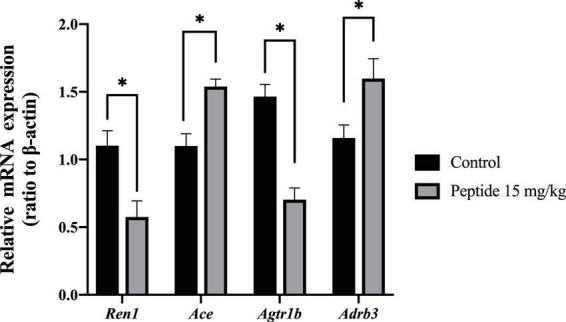
Effect of AEYLCEAC on the expression of ACE related mRNA. *p < 0.05.

After the 4-week of animal trial of AEYLCEAC, expression of genes *Ren1* and *Agtr1* in rats’ kidneys were downregulated, while the expression levels of *Adrb3* were evidently upregulated compared to control (*p < 0.05, Figure 4). However, there was no significant change in ACE gene expression in both. This result is similar to other research which found that *Ren1* expression was downregulated in SHRs orally administered 50 mg/kg of RVPSL hydrolyzed from egg white protein (45). Feng et al. (46) also discovered that gene *Adrb3* expression upregulated by blue mussels (*M. edulis*) peptides at doses of 20 mg/kg in SHRs, which were orally administered. It suggested that peptide AEYLCEAC derived from oysters through GI tract can reduce rats’ blood pressure via multiple gene expression alterations, including downregulation of *Ren1* and *Agtr1* receptor and upregulation of Adrb3.

### Molecular docking analysis

**FIGURE 5:**
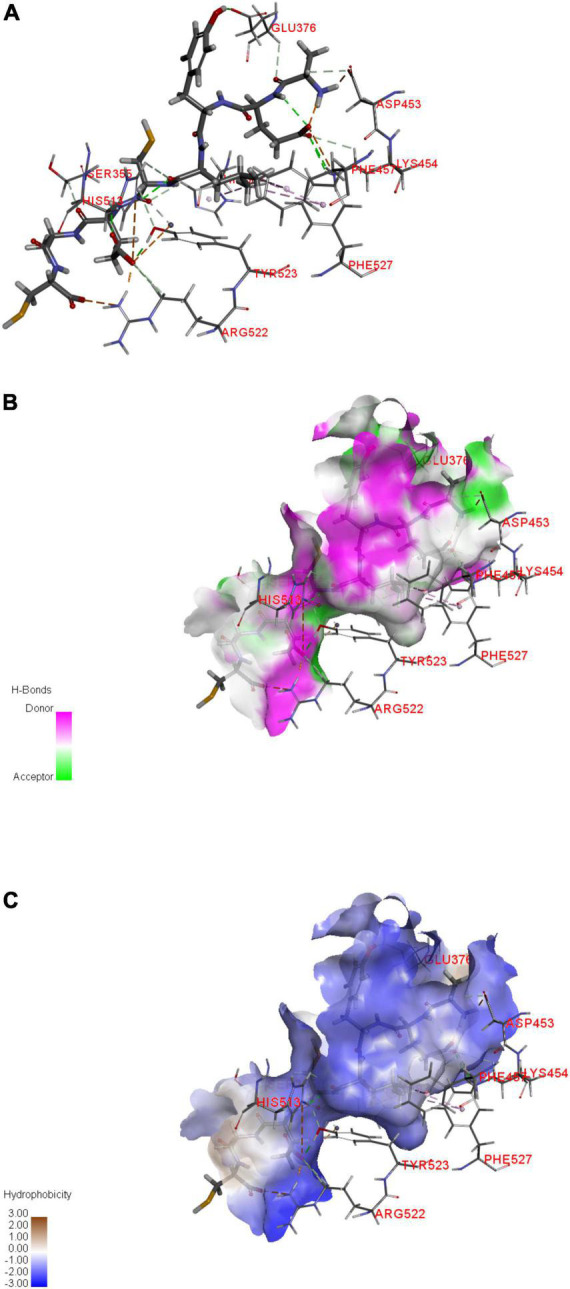
Molecular docking simulations of AEYLCEAC against ACE (PDB: 4CA5). **(A–C)** Represent the active sites, hydrogen bonding, and hydrophobic interactions between AEYLCEAC and ACE, respectively.

Table 3 lists the interaction sites and types of forces between the peptides and ACE. The interaction of peptide with ACE is generated by multiple forces, and the main categories of bonds are carbon-hydrogen bonds, conventional hydrogen bonds, attractive charges, salt bridges, and Pi-Alkyls (47). As shown in Table 3, a total of 18 hydrogen bonds were formed between AEYLCEAC and ACE, including eight residues (Asp453, Lys454, His513, Tyr523, Glu376, Ser355, His383, and Arg522). The dominating interacting residues within the ACE active site are segmented into three pockets (S1, S2 and S1’). S1 contains three key residues including Glu384, Tyr523 and Ala354, and the S2 pocket has five residues as Gln281, His353, Lys511, His513, and Tyr520, while S1’ exhibits only one key residue Glu162 (48). Moreover, the interactions between ACEI peptides and zinc ion (Zn^2+^) often play a pivotal part in the passivation of ACE (49). ACE is a kind of metalloenzyme whose active site had a zinc ion (Zn^2+^), while the Zn^2+^ could interact synergistically with His383, His387, and Glu411 residues (50). Molecular docking analysis showed that AEYLCEAC formed multiple hydrogen bonds in the S1 pocket (Tyr523), S2 pocket (His513), and Zn(II) (His383) of ACE (Figure 5). The residue Arg522 established hydrogen bonds with ACEI peptides derived from potato patatin according to previous literature (51), and peptide AEYLCEAC also formed hydrogen bonds with Arg522 in this study. Additionally, there were four electrostatic interactions between AEYLCEAC and ACE within the ACE residues of Asp453, Lys454, Arg522, and His513. Finally, the electrostatic interaction of Zn (ZN1001) and hydrophobic interactions of His383, Phe457, and Phe527 with ACE were helpful for the binding of the peptide as well. Finally, AEYLCEAC as a whole is hydrophobic, which is due to the fact that E is a hydrophilic amino acid, the other amino acids are hydrophobic or non-polar amino acids. It can fit the hydrophobic area of ACE pocket, indicating that AEYLCEAC can form hydrophobic interaction with ACE. And the H-bonds and the hydrophobility local between AEYLCEAC and ACE are also calculated and shown in filed diagram (Figures 5B,C). The results showed that the inhibition activity of AEYLCEAC may be due to the interactions with ACE active sites, and the peptide could be considered as a potential inhibitor for ACE.

**TABLE 3:** The types of hydrogen bond, electrostatic and hydrophobic interactions of oyster peptide (AEYLCEAC) against ACE.

Interactions	Category	Types
A:ARG522:HH11—AEYLCEAC:O91	Hydrogen bond; electrostatic	Salt Bridge; attractive charge
AEYLCEAC:H2—A:ASP453:OD2	Hydrogen bond; electrostatic	Salt Bridge; attractive charge
A:LYS454:NZ—AEYLCEAC:O25	Electrostatic	Attractive charge
A:HIS513:NE2—AEYLCEAC:O91	Electrostatic	Attractive charge
A:ARG522:NH1—AEYLCEAC:O115	Electrostatic	Attractive charge
A:ZN1001:ZN—AEYLCEAC:O91	Electrostatic	Attractive charge
AEYLCEAC:N1—AEYLCEAC:O25	Electrostatic	Attractive charge
A:LYS454:HZ1—AEYLCEAC:O24	Hydrogen bond	Conventional hydrogen bond
A:LYS454:HZ3—AEYLCEAC:O24	Hydrogen bond	Conventional hydrogen bond
A:HIS513:HE2—AEYLCEAC:O67	Hydrogen bond	Conventional hydrogen bond
A:TYR523:HH—AEYLCEAC:O91	Hydrogen bond	Conventional hydrogen bond
AEYLCEAC:H14—AEYLCEAC:O25	Hydrogen bond	Conventional hydrogen bond
AEYLCEAC:H46—A:GLU376:OE2	Hydrogen bond	Conventional hydrogen bond
AEYLCEAC:H80—AEYLCEAC:O90	Hydrogen bond	Conventional hydrogen bond
A:SER355:HB1—AEYLCEAC:O93	Hydrogen bond	Carbon hydrogen bond
A:GLU376:HA—AEYLCEAC:O12	Hydrogen bond	Carbon hydrogen bond
A:HIS383:HD2—AEYLCEAC:O78	Hydrogen bond	Carbon hydrogen bond
A:LYS454:HE2—AEYLCEAC:O24	Hydrogen bond	Carbon hydrogen bond
A:HIS513:HE1—AEYLCEAC:O67	Hydrogen bond	Carbon hydrogen bond
A:ARG522:HD1—AEYLCEAC:O91	Hydrogen bond	Carbon hydrogen bond
A:ARG522:HD2—AEYLCEAC:O91	Hydrogen bond	Carbon hydrogen bond
AEYLCEAC:H6—A:ASP453:OD2	Hydrogen bond	Carbon hydrogen bond
AEYLCEAC:H71—A:TYR523:OH	Hydrogen bond	Carbon hydrogen bond
A:ZN1001:ZN—AEYLCEAC:O78	Other	Metal-Acceptor
A:HIS383—AEYLCEAC:C62	Hydrophobic	Pi-Alkyl
A:PHE457—AEYLCEAC:C58	Hydrophobic	Pi-Alkyl
A:PHE527—AEYLCEAC:C58	Hydrophobic	Pi-Alkyl
A:PHE527—AEYLCEAC:C62	Hydrophobic	Pi-Alkyl

### Secondary structure alteration of angiotensin-I-converting enzyme affected by AEYLCEAC

**FIGURE 6:**
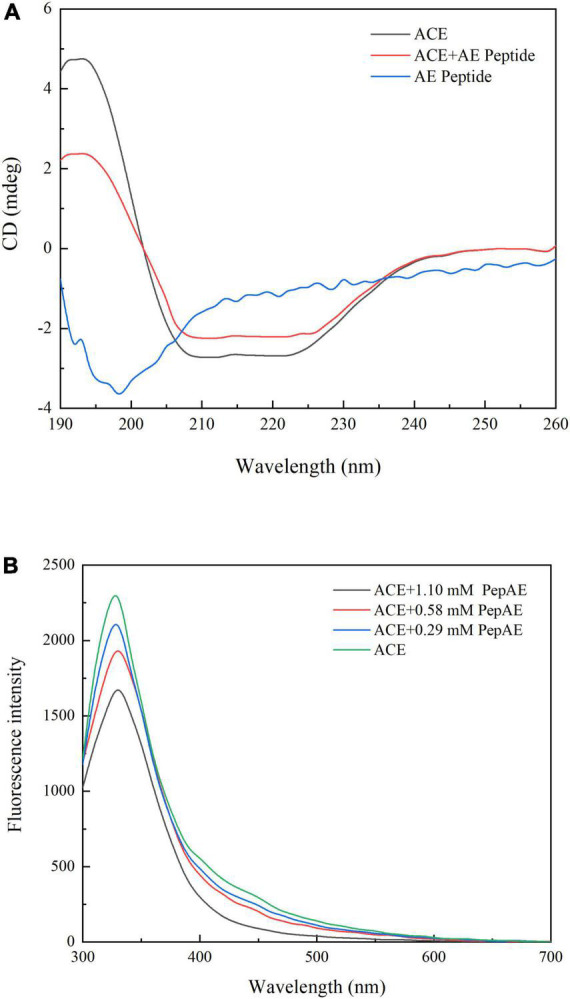
**(A)** Far-UV CD spectra results of the peptide AEYLCEAC as well as ACE in the absence or presence of AEYLCEAC. The mass ratio of ACE: peptide was about 7:1. **(B)** Fluorescence emission spectra results of ACE with increasing concentrations of peptide AEYLCEAC (0.29, 0.58, 1.1 mM).

Circular dichroism spectrum (CD) is an effective way to investigate the interactions between inhibitors and target enzyme since the 1960s (52). As shown in Figure 6, it is evident that the multiple interactions between AEYLCEAC and ACE generate the transformation of the helix content at the wavelengths of 208 nm (π–π*) and 222 nm (n–π*) (53). The compositions of secondary structure consisted of 38.2% α-helix, 15.5% β-sheet, 16.2% turn, and 30.1% unordered coil (see Table 4). The interaction between AEYLCEAC and ACE causes significant changes in ACE spectral characteristics. The results showed that the α-helical structure of the ACE-AEYLCEAC complex decreased from 38.2 to 24.1%, while the total β-structure and unordered structure increased by 8.5 and 5.6%, respectively. The results suggest that there is a partial unfolding of ACE. The reduction of α-helix and the raise of an unordered coil in the protein complex possibly lead to the decrease of hydrogen bonding as well as exposures of the hydrophobic cavities (54). The secondary structure contents determine the tertiary structure of the protein, so it is closely related to the biological activity of the enzyme (55). Thus, these findings demonstrated that the secondary structure content of ACE is changed by the addition of peptide AEYLCEAC.

**TABLE 4:** Secondary structure change of ACE with peptide analyzed by CD.

System	α -helix (%)	β -turn (%)	Antiparallel (%)	Parallel (%)	Random coil (%)
ACE	38.2	16.2	7.6	7.9	30.1
ACE-Peptide	24.1	17.8	12.4	10	35.7

Beside, we also study the peptide binding to ACE using a fluorescence spectroscopy (56). AEYLCEAC had an emission maximum at 330 nm upon the excitation of 270 nm. As the concentrations of the synthetic peptide increased, the fluorescence intensity of ACE decreased remarkably, indicating the synthetic peptide AEYLCEAC could induce fluorescence quenching and interacted with ACE (Figure 6B). According to the previous report, when the excitation wavelength is 270 nm, the ACE fluorescence mainly came from Trp and Tyr, which suggested that the microenvironment of Trp and Tyr residues may have changed (54). The different quenching mechanisms are generally divided into two types: static and dynamic quenching (57). Based on the fluorescence spectra, the quenching rate constant (*K*_*q*_) was determined by the Stern-Volmer equation. And the *K*_*q*_ values of all three concentrations of AEYLCEAC were larger than 2 × 10^10^ M^–1^.s^–1^ (the maximum diffusion collision quenching constant), which might be related to static quenching. In conclusion, the results illustrated that the synthetic peptide showed a binding capacity with ACE and can effectively quench the intrinsic fluorescence.

## Conclusion

In this study, a novel ACEI peptide AEYLCEAC was screened from oyster hydrolysate and synthesized, which can resist digestive enzyme degradation. The peptide has a competitive inhibitory effect on ACE substrates, and can effectively reduce SBP and DBP of hypertensive rats after the short-term and long-term oral intervention. The long-term implications of this research are the ACEI peptide AEYLCEAC from oysters could be served as a useful bioactive peptide, which is expected to be further developed and utilized as a functional food ingredient for the remission and treatment of hypertension on an industrial scale. Nevertheless, further experiments need to be carried out to examine the ACEI activity of AEYLCEAC *in vivo*.

## Data availability statement

The original contributions presented in this study are included in the article/Supplementary material, further inquiries can be directed to the corresponding author/s.

## Ethics statement

The animal study was reviewed and approved by the Ethics Committee on Animal Experimentation of Zhejiang University of Technology (Permission Number 20210309043).

## Author contributions

HC, XX, and YC provided the project administration and funding acquisition. HZ designed the research and wrote the manuscript. HZ, YC, and LX executed the experiments and analyzed the data. HC, XX, and LX reviewed and edited this manuscript. All authors have read and agreed to the published version of the manuscript.

## Footnotes

1
https://www.rcsb.org/
