# Supplementary material for: A novel angiotensin-I-converting enzyme inhibitory peptide from oyster: Simulated gastro-intestinal digestion, molecular docking, inhibition kinetics and antihypertensive effects in rats
Source: Front Nutr. 2022 Aug 23;9:981163. doi: 10.3389/fnut.2022.981163 (PMC9445672; doi:10.3389/fnut.2022.981163)
Supplement: Supplementary file 1 [file Data_Sheet_1.PDF]

Supplementary data information description

Table S1 Primers desined for Rt-PCR

Fig. S1 HPLC results of the potential ACEI oyster peptide screened by molecular docking simulation: (A) RSNDGPI; (B) VILGDADLP; (C) TNEVEGPS; (D) LDVSWASD; (E) QEVVMGEC; (F) LSSNLHG.

Fig. S2 MS spectrum of the potential ACEI oyster peptide screened by molecular docking simulation: (A) RSNDGPI; (B) VILGDADLP; (C) TNEVEGPS; (D) LDVSWASD; (E) QEVVMGEC; (F) LSSNLHG.

Table S1 Primers desined for Rt-PCR

**Gene**	**Forward Primer (5' to 3')**	**Reversed Primer (5' to 3')**	**length (bp)**
β-Actin, (*Actb*)	ATTGTTACCAACTGGGACGACATG	CAGCCTGGATGGCTACGTACATG	193
Renin 1 structural (*Ren1*)	GTCCTGTGGGTGTGTATA	GAGCAAGATTCGTCCAAA	175
Angiotensin I converting enzyme (peptidyldipeptidase A) 1 (*Ace*)	CACCGGCAAGGTCTGCTT	CTTGGCATAGTTTCGTGAGGAA	55
Angiotensin II receptor, type 1b (*Agtr1b*)	CGGCCTTCGGATAACATG	CTGTCACTCCACCTCAAAACA	67
Adrenergic receptor, β3 (*Adrb3*)	ATCTTAGCCAGGATTGAGGTGGAG	AGTTACCCGGAGACACATGAGGA	85

Fig. S1

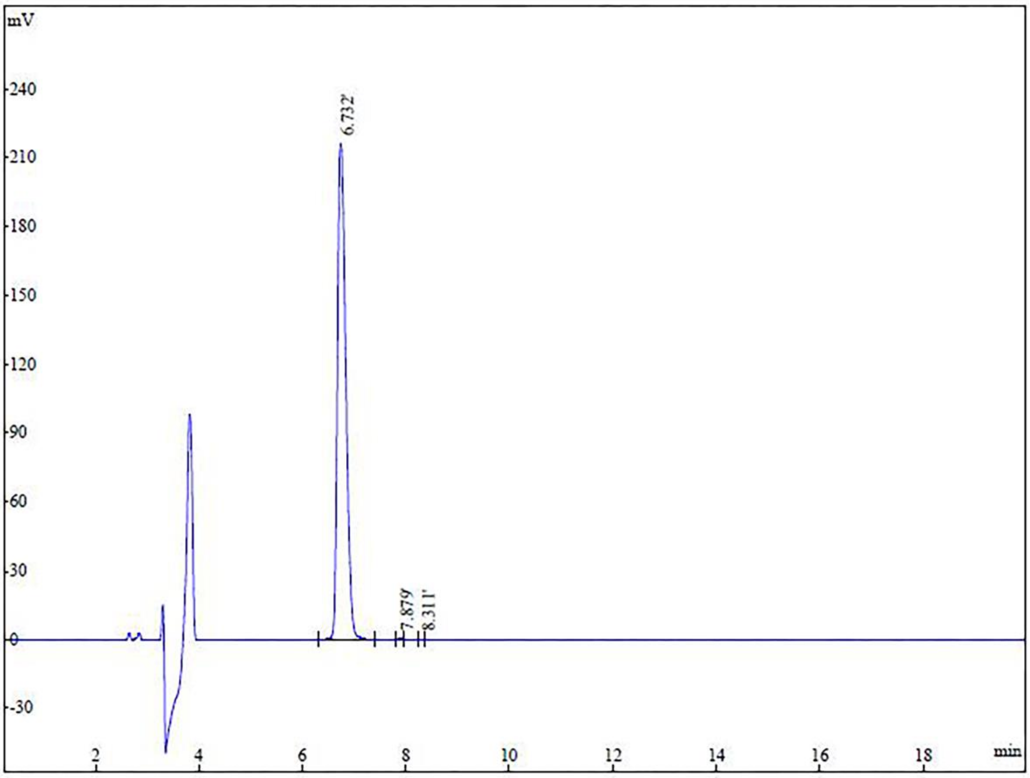

mV
240
210
180
150
120
90
60
30
0
-30
6.732
7.879
8.311
2
4
6
8
10
12
14
16
18
min

(A)

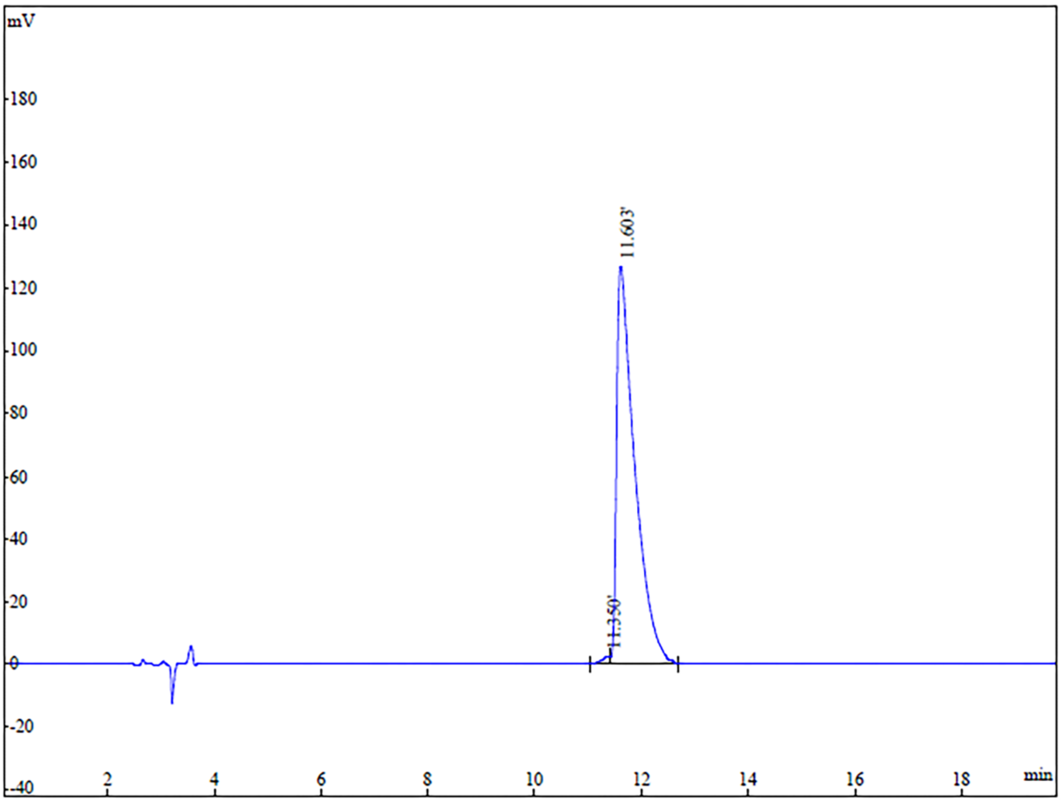

mV
180
160
140
120
100
80
60
40
20
0
-20
-40
11.603
11.350
2
4
6
8
10
12
14
16
18
min

(B)

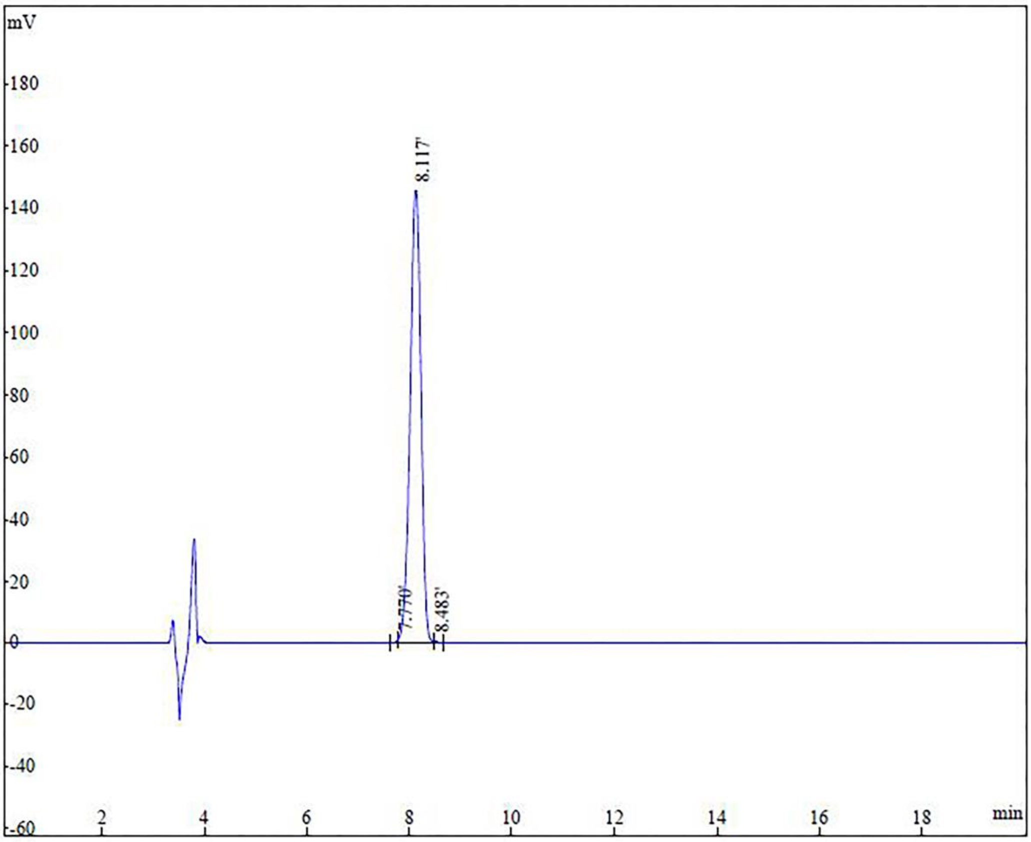
mV
180
160
140
120
100
80
60
40
20
0
-20
-40
-60
8.117'
7.770'
8.483'
2
4
6
8
10
12
14
16
18
min

(C)

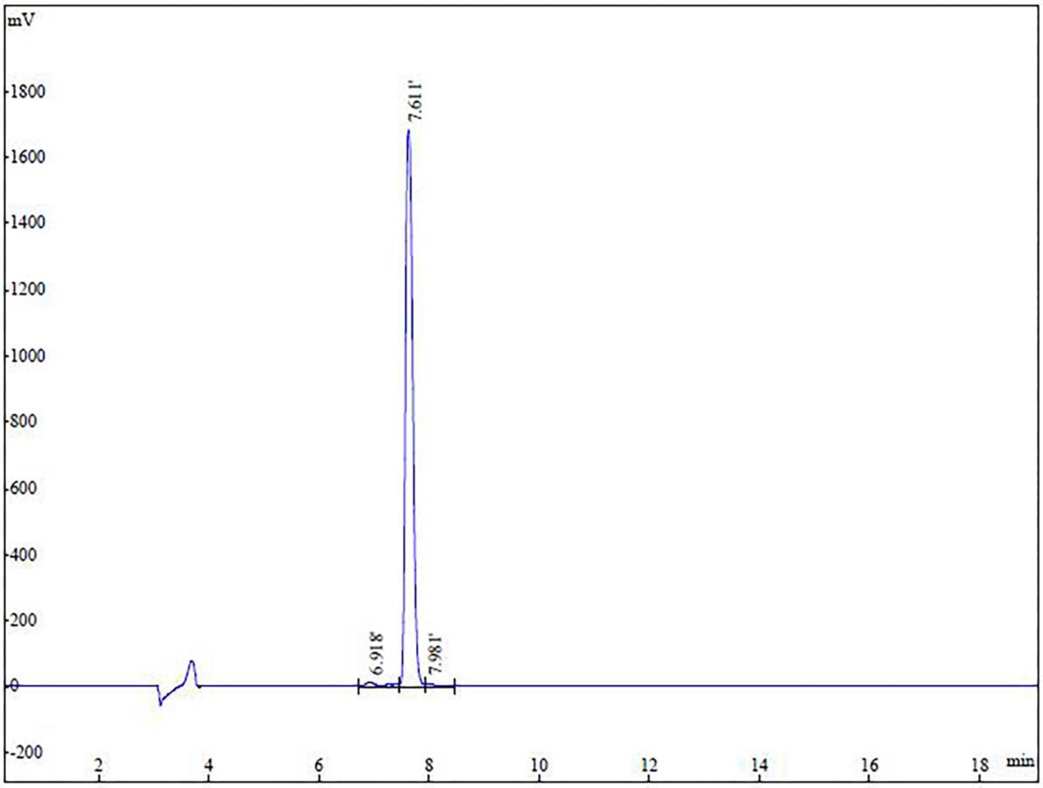
mV
1800
1600
1400
1200
1000
800
600
400
200
0
-200
7.611'
6.918'
7.981'
2
4
6
8
10
12
14
16
18
min

(D)

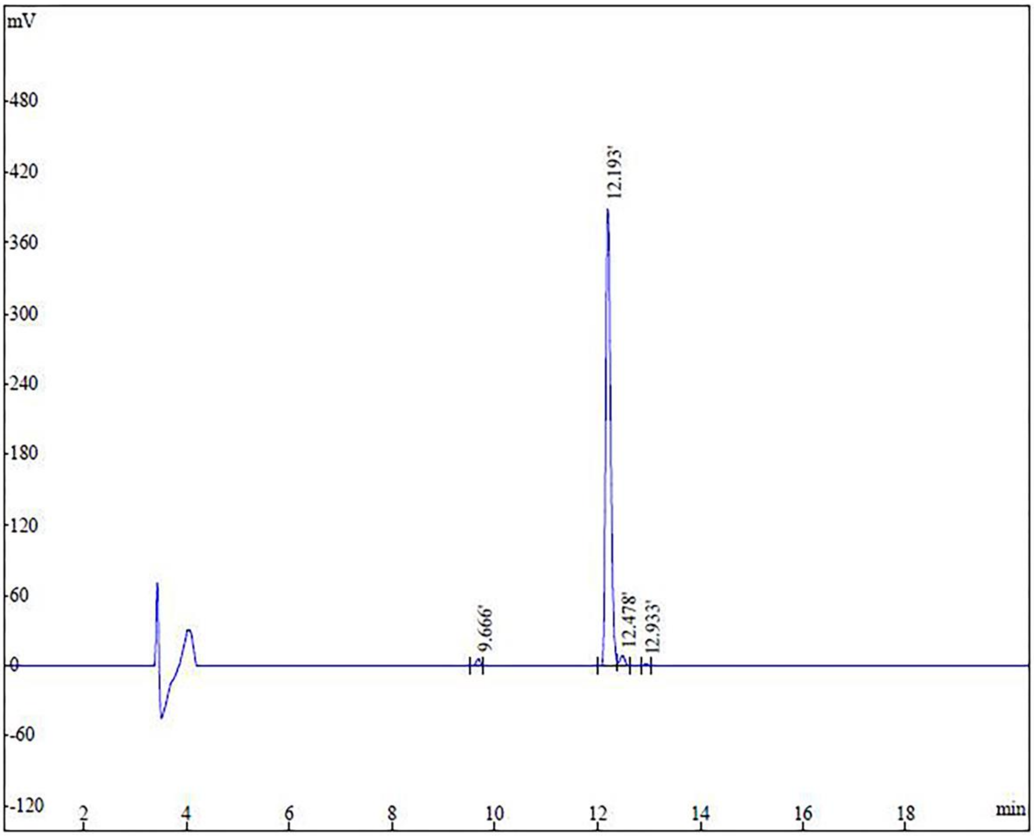
mV
480
420
360
300
240
180
120
60
0
-60
-120
2
4
6
8
10
12
14
16
18
min
12.193'
9.666'
12.478'
12.933'

(E)

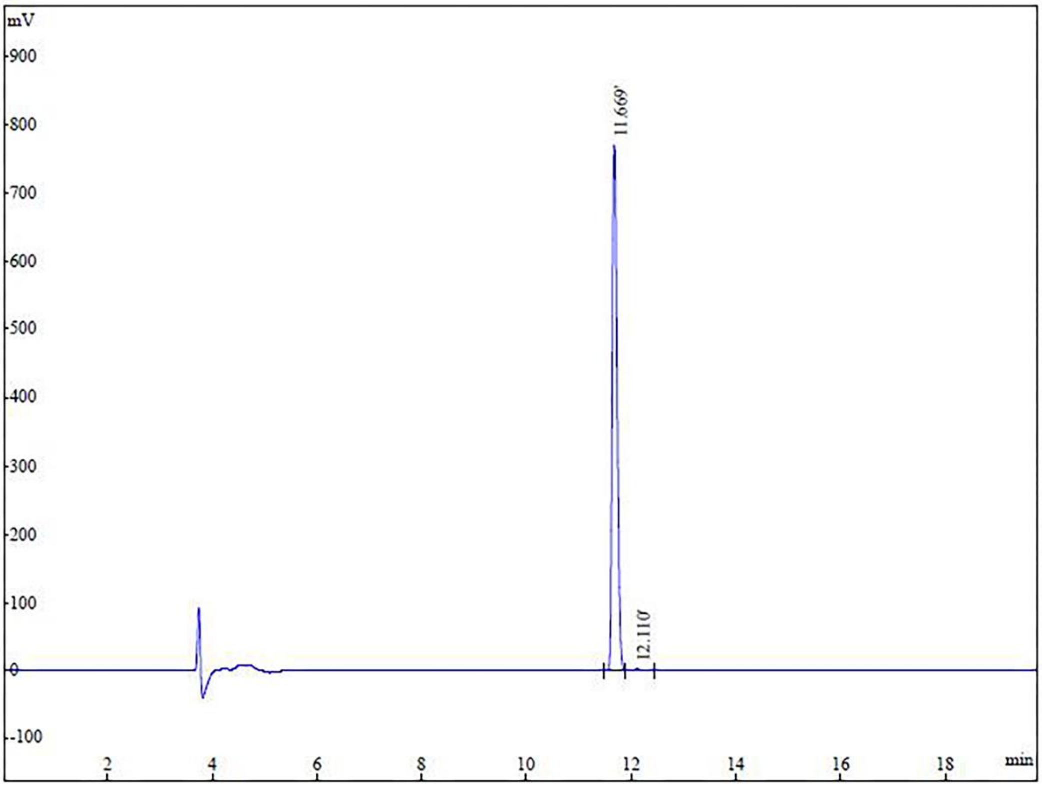
mV
900
800
700
600
500
400
300
200
100
0
-100
2
4
6
8
10
12
14
16
18
min
11.669'
12.110'

(F)

Fig. S2

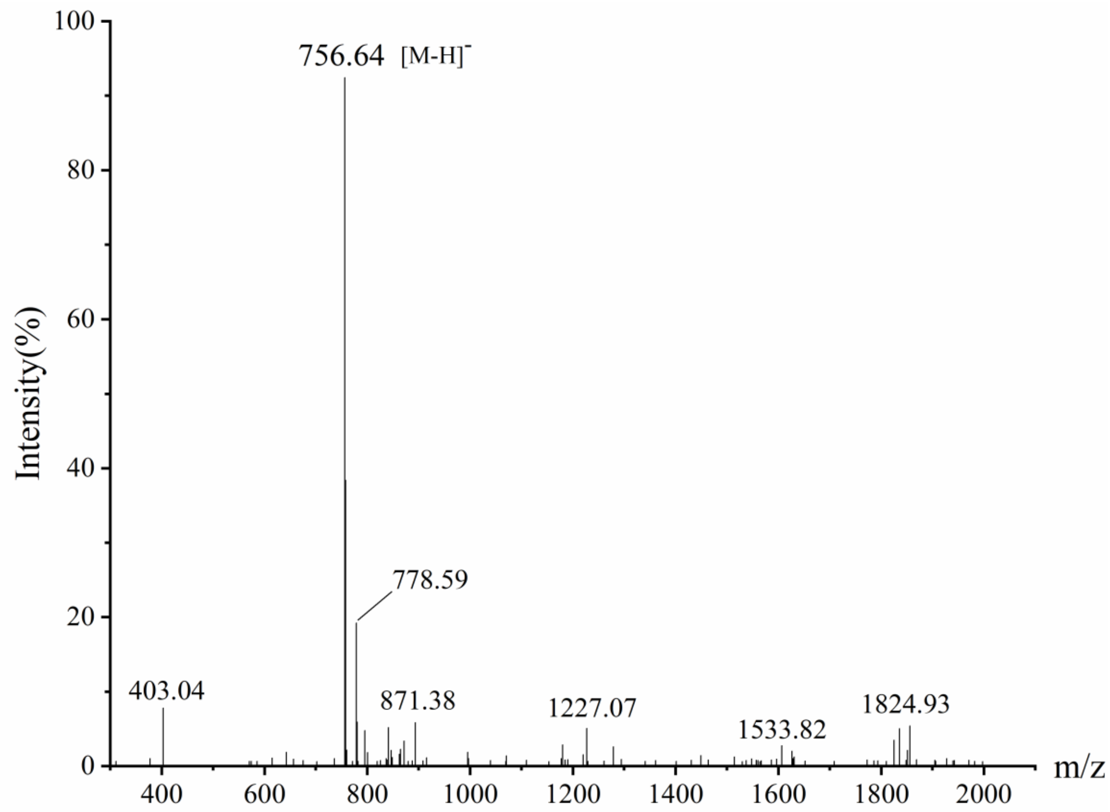

756.64 [M-H]⁻
778.59
403.04
871.38
1227.07
1533.82
1824.93
Intensity(%)
m/z
100
80
60
40
20
0
400
600
800
1000
1200
1400
1600
1800
2000

(A)

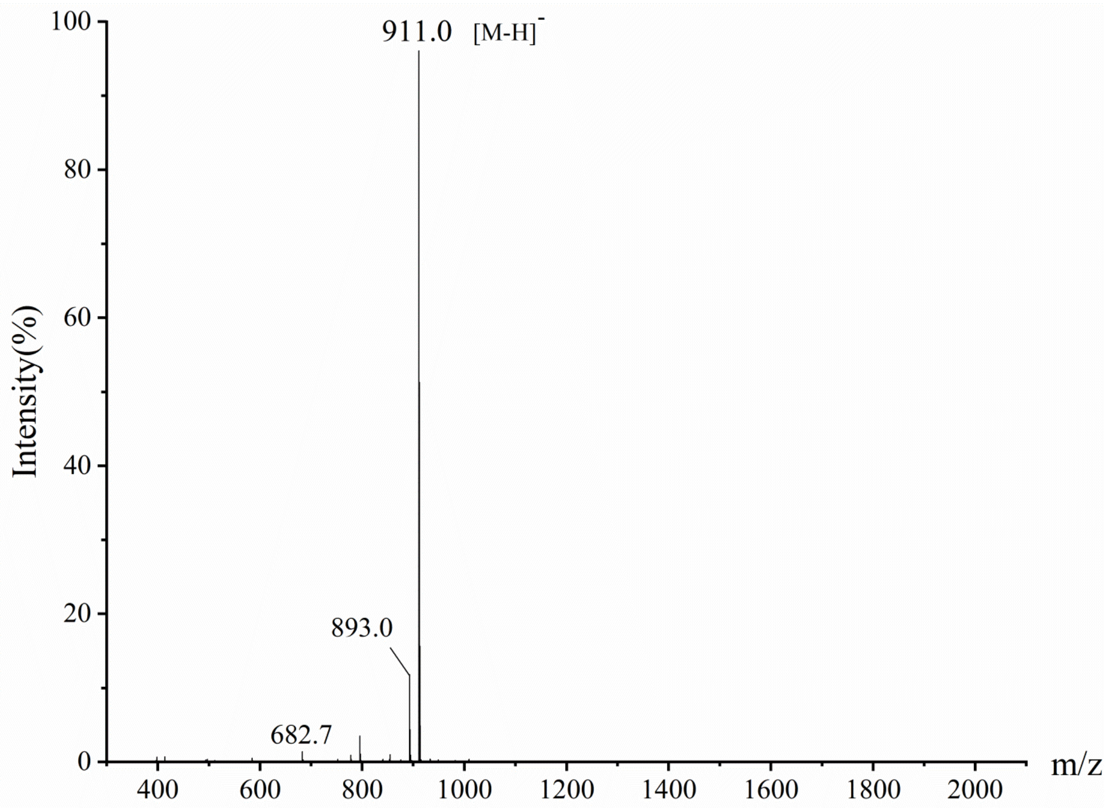
911.0 [M-H]-
893.0
682.7
Intensity(%)
m/z
100
80
60
40
20
0
400
600
800
1000
1200
1400
1600
1800
2000

(B)

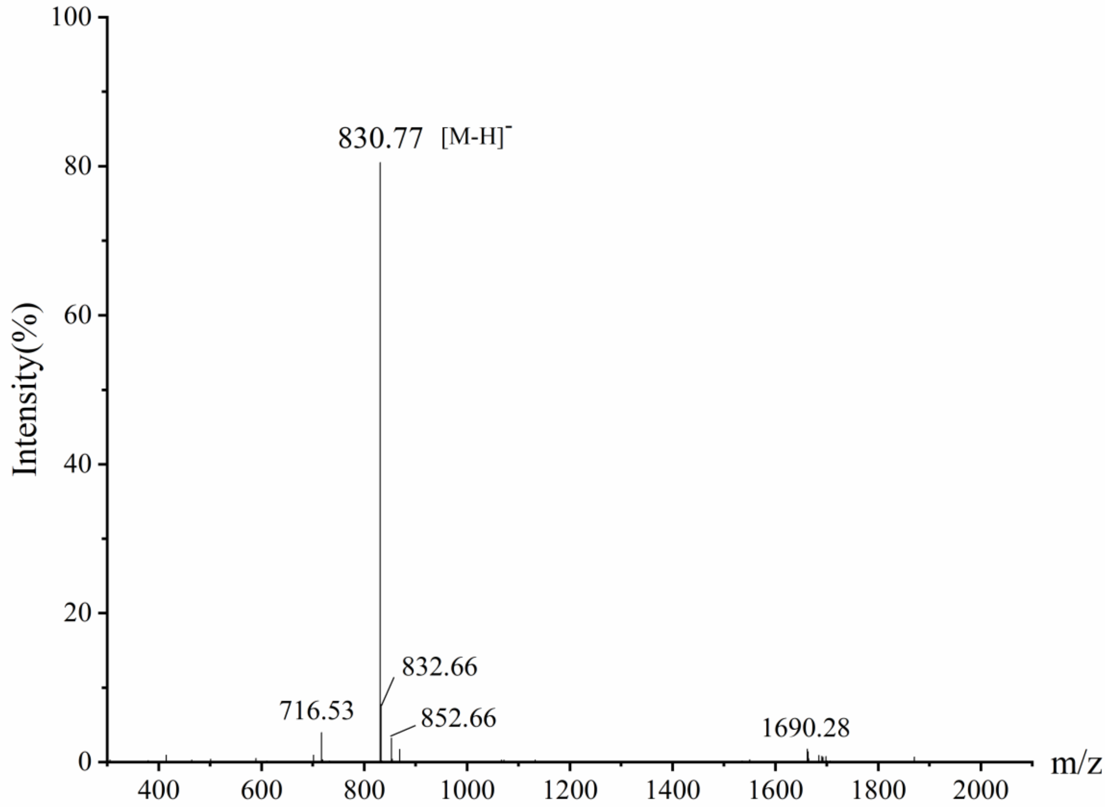
830.77 [M-H]-
832.66
852.66
716.53
1690.28
Intensity(%)
m/z
100
80
60
40
20
0
400
600
800
1000
1200
1400
1600
1800
2000

(C)

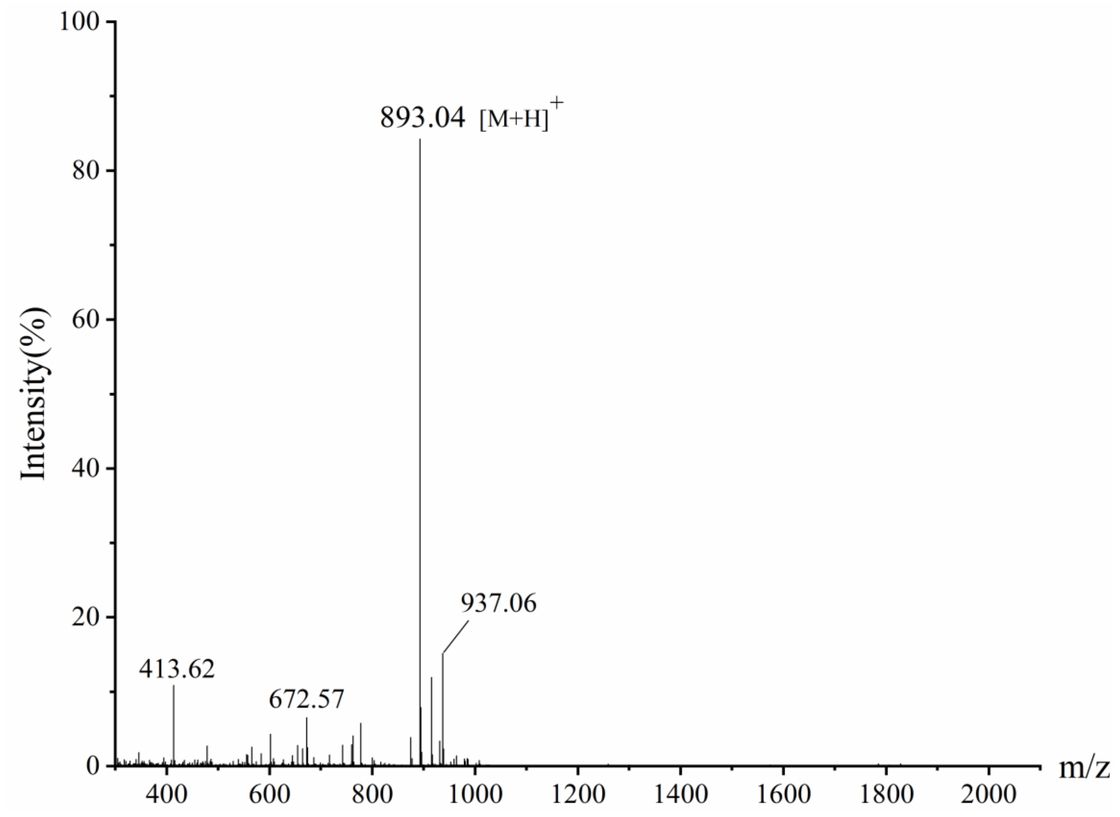
100
80
60
40
20
0
Intensity(%)
893.04 [M+H]+
937.06
413.62
672.57
400
600
800
1000
1200
1400
1600
1800
2000
m/z

(D)

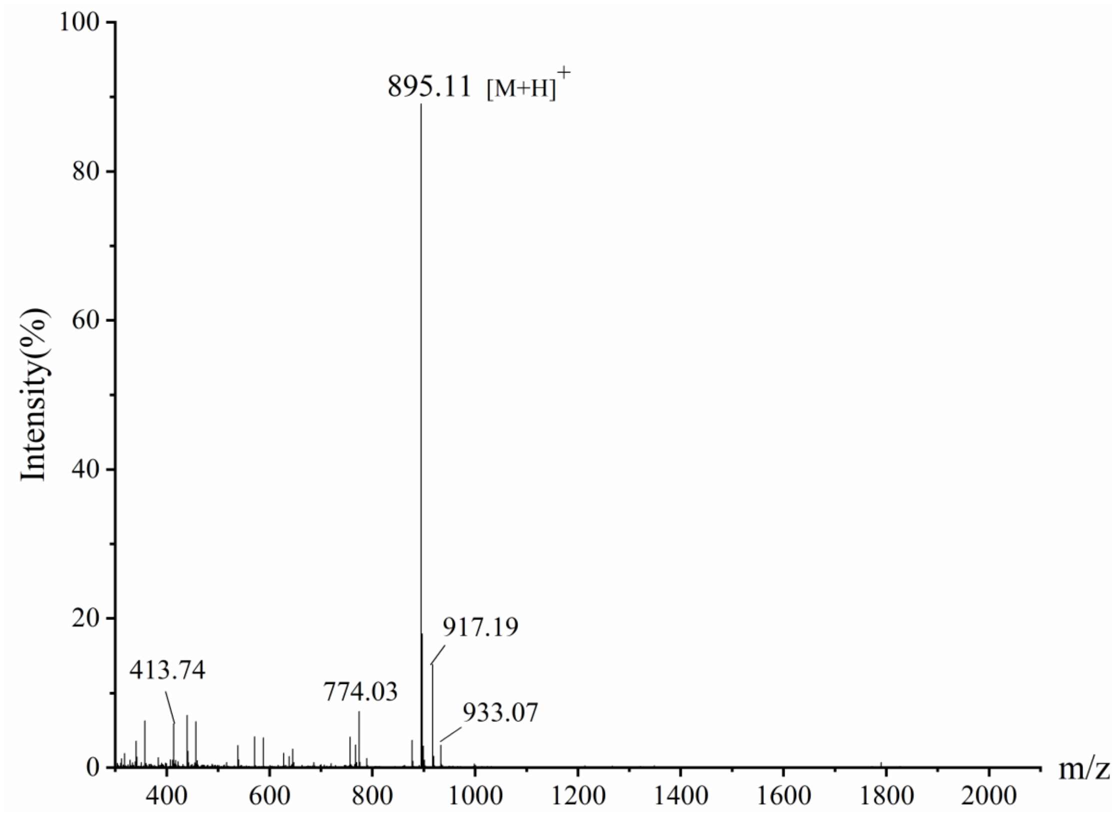
100
80
60
40
20
0
Intensity(%)
895.11 [M+H]+
917.19
413.74
774.03
933.07
400
600
800
1000
1200
1400
1600
1800
2000
m/z

(E)

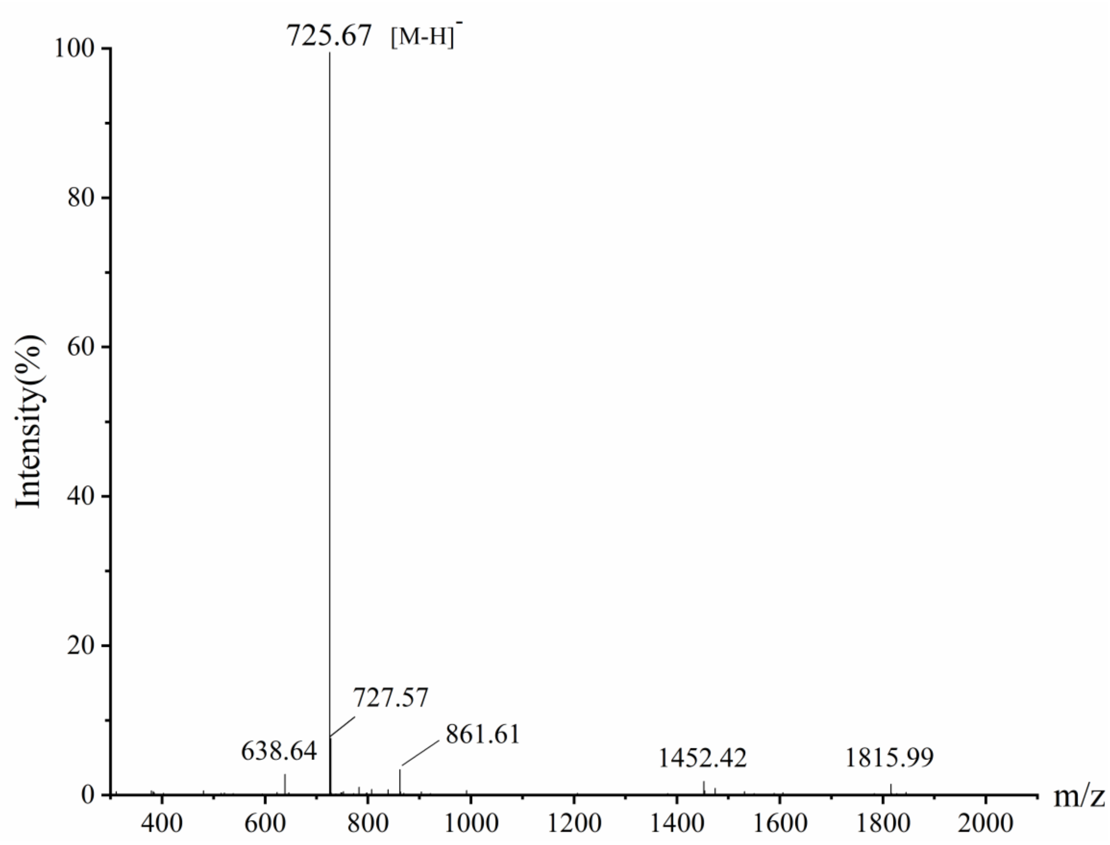
725.67 [M-H]-
100
80
60
40
20
0
Intensity(%)
727.57
638.64
861.61
1452.42
1815.99
m/z
400
600
800
1000
1200
1400
1600
1800
2000

(F)
